# Learning curve: machine learning takes on pediatric hematologic malignancy risk-stratification

**DOI:** 10.1038/s41390-024-03661-y

**Published:** 2024-10-25

**Authors:** Zoe King, William B. Mitchell

**Affiliations:** Department of Pediatrics, Albert Einstein College of Medicine, Bronx, NY USA

Machine learning (ML) is a subcategory of artificial intelligence that focuses on pattern recognition in large data sets. ML evaluates large data sets and associates complex variables with outcomes, essentially performing risk stratification. This ML function dovetails with the needs of pediatric oncology, in which risk stratification has played a pivotal role in improving the outcomes of pediatric malignancies over the last 60 years.^1–3^ This is especially the case for pediatric hematologic malignancies, some of which now have survival rates of over 90%. Early on, the treatments for pediatric hematologic malignancies were largely uniform, using similar regimens for all patients. As clinicians began to recognize clinical features of acute lymphoblastic leukemia that were associated with worse or better outcomes, they were able to use more therapy for higher-risk patients and less for lower-risk patients. This resulted in both increased overall survival and decreased toxicity for lower-risk patients. In myeloid malignancies the discovery of cytogenetic abnormalities associated with different risk groups allowed further risk stratification, leading to more specific therapies for the different risk groups. Further advances in molecular biology led to the discovery of numerous specific gene mutations that allowed even more nuanced risk stratification for almost all patients with hematologic malignancies. Most recently, response to therapy, in the form of minimal residual disease after induction chemotherapy, has become an important risk marker and has significant implications for therapy and survival.

The application of ML in the risk stratification of pediatric leukemia is an evolving field. While traditional risk stratification methods rely on well-established clinical and biological factors, ML offers the ability to analyze vast datasets in an unbiased way to uncover nuanced patterns that may elude conventional statistical approaches. By integrating ML into risk stratification, researchers can potentially identify new biomarkers, improve predictions of patient outcomes with greater accuracy, and tailor therapies more precisely to individual patients. This review by Gurumurthy et al. probes the current state of ML risk-stratification by performing a comprehensive systematic review of the current literature. The authors included twenty studies in their final analysis, primarily in hematological malignancies. Their data show moderate to good predictive capabilities for prognosis, treatment-response and toxicity algorithms. This is a strong start for ML in the pediatric oncology risk stratification field.

The ability of ML to develop algorithms is only as strong as the data it is given to analyze. Most of the ML studies that were not included in the analysis had important limitations, namely small sample size, lack of standardized data entry and reporting, and infrequent use of external validation. Use of larger databases and establishment of common data models between large databases to train, test, and validate ML algorithms will significantly advance the field. By utilizing large multi-center data sets, researchers could train ML algorithms on a broader spectrum of patient characteristics, enhancing the robustness and accuracy of risk stratification models. This could be accomplished by large national or international oncology organizations. As with other database-driven research, standardization of terms and reporting will be crucial to the applicability of such an effort. With this systematic review, Gurumurthy et al. have presented a snapshot in time of the beginnings of the field of ML-driven risk stratification of pediatric malignancies: there will certainly be more to come.
